# Effects of a single administration of prostaglandin F2alpha, or a combination of prostaglandin F2alpha and prostaglandin E2, or placebo on fertility variables in dairy cows 3–5 weeks post partum, a randomized, double-blind clinical trial

**DOI:** 10.1186/1477-7827-4-65

**Published:** 2006-12-21

**Authors:** Gaby Hirsbrunner, Heinz W Burkhardt, Adrian Steiner

**Affiliations:** 1 Clinic for Ruminants, Vetsuisse Faculty of the University of Berne, Bremgartenstrasse 109a, 3012 Berne, Switzerland; 2 Dr. E. Gräub AG, 3018 Berne, Switzerland

## Abstract

**Background:**

Delayed uterine involution has negative effects on the fertility of cows; use of prostaglandin F2alpha alone as a single treatment has not been shown to consistently improve fertility. Combined administration of PGF2alpha and PGE2 increased uterine pressure in healthy cows. We hypothesized, that the combination of both prostaglandins would accelerate uterine involution and have, therefore, a positive effect on fertility variables. In commercial dairy farming, the benefit of a single post partum combined prostaglandin treatment should be demonstrated.

**Methods:**

383 cows from commercial dairy farms were included in this study. Uterine size and secretion were evaluated at treatment 21–35 days post partum and 14 days later. Cows were randomly allocated to one of three treatment groups: PGF2alpha and PGE2, PGF2alpha or placebo. For every animal participating in the study, the following reproduction variables were recorded: Interval from calving to first insemination, days open, number of artificial inseminations (AI) to conception; subsequent treatment of uterus, subsequent treatment of ovaries. Plasma progesterone level at time of treatment was used as a covariable. For continuous measurements, analysis of variance was performed. Fisher's exact test for categorical non-ordered data and exact Kruskal-Wallis test for ordered data were used; pairwise group comparisons with Bonferroni adjustment of significance level were performed.

**Results:**

There was no significant difference among treatment groups in uterine size. Furthermore, there was no significant difference among treatments concerning days open, number of AI, and subsequent treatment of uterus and ovaries. Days from calving to first insemination tended to be shorter for cows with low progesterone level given PGF2alpha and PGE2 in combination than for the placebo-group (P = 0.024).

**Conclusion:**

The results of this study indicate that the administration of PGF2alpha or a combination of PGF2alpha and PGE2 21 to 35 days post partum had no beneficial effect upon measured fertility variables. The exception was a tendency for a shorter interval from calving to first insemination after administration of the combination of PGF2alpha and PGE2, as compared to the placebo group. Further research should be done in herds with reduced fertility and/or an increased incidence of postpartum vaginal discharge.

## Background

Delayed uterine involution causes economic losses to dairy farmers. Uterine involution is normally completed between 26 to 52 days post partum, but changes after 20–25 days are often imperceptible [1]. Puerperal controls are often done between 20–33 days post partum [2,3]. This period overlaps with the period for normal postpartum involution, but this window of time corresponds to the period when post partum controls were described in German [4] and Canadian studies [5]. First visit criteria include evaluation of condition and involution of the uterus and type of vaginal discharge [6,7]. Reevaluation can be done 14 days later [2,8,9].

Inadequate production of endogenous prostaglandin has been associated with delay in uterine involution post partum [10,11]. Repeated administration of prostaglandin F_2α _(PGF_2α_) twice daily from days 3 to 13 after calving shortened the time needed for uterine involution by 6 days [12]. In the early post partum period, even single administration of PGF_2α _does accelerate uterine involution and hasten a return to fertile ovarian cyclicity [10,12-14]. Single administrations of PGF_2α _14 to 28 days post partum resulted in an enhanced first service conception rate (68% as compared to 43% in control cows) [15]. The advantages of this therapy, though, were restricted to herds with below average conception rates (< 50% to first insemination) [13]. Improved conception rate after single administration of dinoprost in cows 14 to 28 days post partum was particularly seen in cows with a serum progesterone value less than 0.5 ng/ml [15]. This fact demonstrated that the benefit of a prostaglandin therapy was not the consequence of luteolysis [15,16], but the beneficial effect of PGF_2α _administered post partum is rather the result of myometrial contraction and thereby accelerated uterine involution [17,18].

In healthy experimental cows, prostaglandin E2 (PGE2) increased uterine pressure in a dose-dependent manner if administered intravenously [19]. Furthermore, there was a cumulative effect related to uterine pressure, if PGF_2α _and PGE2 were given in combination [20]. In humans, the potency of prostaglandin E2 in stimulating smooth muscle contractility exceeded that of PGF_2α _[21], whereas the two substances were equally potent as to myometrial contractility in cows near parturition [22]. In experimental cows, increased uterine pressure of PGE2 compared to PGF_2α _was only measurable within the first 15 min after administration [20]. Obviously, there are two different receptors for PGE2 and PGF_2α_, but they also seem to be able to bind to the same receptor with different affinities [21,23]. PGE2 may even bind to the PGF_2α _– receptor with higher affinity than PGF_2α _itself [21] and furthermore, PGE2 is degraded via PGF_2α _[24]. In humans, it was, furthermore, suggested that a greater number of PGE receptors was responsible for the greater potency for PGE's in stimulating uterine contractions compared to that of PGF_2α _[25]. We decided to use a d-cloprostenol (as to PGF_2α _: there are copies of the natural PGF_2α _and synthetic more potent cloprostenols which are racemic or in the d-form), as it can be used in a lower dose than the dl-cloprostenol, and it has an equal effect on myometrial contractility [26].

It was the purpose of our study to evaluate the effect of a single administration of PGF_2α_, or PGF_2α _and PGE2 in combination (= PGF_2α _+ E2), or placebo in dairy cows 21 to 35 days post partum. Our hypothesis was, that the combination of both PG's had a beneficial effect on measured fertility variables based on accelerated uterine involution and cycle induction.

## Materials and methods

### Animals and including criteria

Data from 80 herds (10 – 50 cows per herd) participating in the Fertility Service of the Clinic for Ruminants, Vetsuisse Faculty of the University of Berne were evaluated (biweekly herd visits). Dairy cows undergoing routine puerperal control 21 to 35 days post partum were included, except for cows treated for puerperal endometritis, cows with preceding caesarean section, retained foetal membranes, dystocia, systemic antibiotic treatment and systemic illnesses such as ketosis, displacement of the abomasum, or acute mastitis. Furthermore, cows under suspicion of not being healthy by the farmer were excluded of the study because in these cases, the farmers had called their veterinarian before our herd health visit to treat these cows.

### Study design

All trials were performed as double blind trials by informed consent of the farmer (neither investigators nor cows' owners were informed as to the group of treatment used). Before drug administration, a blood sample was taken for progesterone analysis later. Plasma progesterone analysis was performed using an enzymeimmunoassay with the second antibody technique. [27,28]. Every farm was an entity starting systematically with treatment "A" for the first cow, followed by "B", "C", "A", "B" and so on.

### Treatment

Three groups of treatments were used: A = combination of PGF_2α _(Genestran^®^, Dr. E. Gräub AG, Berne Switzerland, 150 μg d-cloprostenol IV) and PGE2 (2.5 mg dinoprostone IV), both substances administered in a volume of 2 ml each; B = placebo (NaCl 0.9% IV) in 2 portions of 2 ml; C = PGF_2α _(Genestran^®^, 150 μg d-cloprostenol IV) and placebo (NaCl 0.9% IV), both substances in 2 portions of 2 ml.

### Clinical variables

Cows were clinically examined by rectal and vaginal exploration and vaginoscopy by one of four different trained investigators working in herd health management. All investigators received standard instructions before starting work in the Fertility Service. Uterine size and location, texture of uterine wall, symmetry of uterine horns, cervical diameter, structures on ovaries and quantity and quality of uterine secretion were recorded in a routine manner. Within this study only uterine size and secretion were analyzed for this study.

#### Uterine size

During rectal palpation, uterine size was scored as "under 1 examinators' hand", "pelvic", "abdominal-delimitable" or "abdominal-not delimitable" [29].

#### Uterine secretion

Uterine secretion scored during vaginoscopy was categorized as "normal", including clear mucus with some flakes of pus or "purulent" meaning more than 50% of pus or "lochiae" (reddish, brown, not malodorous) as reported in previous studies [2,5,6,30].

### Clinical reevaluation, side effects

All animals included in the study were clinically reevaluated by vaginal and rectal palpation and vaginoscopy 14 days after the first clinical examination and treatment by the same investigator. The same clinical variables were evaluated and recorded similar to the first examination.

Owners were requested for recording any adverse side effects following treatment and the injection site was monitored during reevaluation.

### Definitions

The changing of uterine size from first to second evaluation was categorized as physiologic, healed or delayed (Table 1).

**Table 1:** Definition of progress from first examination of uterine size by rectal palpation 21 to 35 days post partum to second examination 14 days later.

**First examination**	**Second examination**	**Definition of progress**
under 1 examiners hand	under 1 examiners hand	physiologic
pelvic	under 1 examiners hand	healed
abdominal-delimitable	under 1 examiners hand	healed
abdominal-not delimitable	under 1 examiners hand	healed
all other combinations		delayed

The changing of uterine secretion was scored as physiologic, healed or pathologic (Table 2).

**Table 2:** Definition of progress from first examination 21 to 35 days post partum to second examination 14 days later of uterine secretion.

**First examination**	**Second examination**	**Definition of progress**
normal	normal	physiologic
purulent	normal	healed
lochiae	normal	healed
all other combinations		pathologic

'Normal' means clear or clear with some flakes of pus. 'Purulent' means more than 50% of pus, 'lochiae' means reddish brown discharge, not malodorous.

#### Fertility variables

Reproductive management in the herds included insemination on the first oestrus observed after a voluntary waiting period of 42 days.

For every animal participating in the study, the following variables were recorded: Interval from calving to first insemination, days open, number of AI to conception, subsequent treatment of uterus (local administration of antibiotics or disinfectants); subsequent treatment of ovaries (PGF_2α_, progesterone-releasing intravaginal devices, GnRH for the following reasons: cysts, not showing heat, persistent corpus luteum (CL)).

#### Covariables

Plasma progesterone level on the day of drug administration was used as a covariable. Definition of "with CL" was a palpable corpus luteum and a serum progesterone value > 2 ng/ml.

### Statistical analyses

Statistical analyses were performed with StatXact 6.0 (Cytel Inc., Cambridge MA, USA). For continuous measurements, analysis of variance was performed. Fisher's exact test for categorical non-ordered data and exact Kruskal-Wallis test for ordered data were used; pairwise group comparisons with Bonferroni adjustment of significance level were performed. Level of significance was p = 0.050 and with Bonferroni adjustment p < 0.017. All data were calculated irrespective of progesterone value and grouped in "with corpus luteum" or "without corpus luteum".

## Results

### Animals

Data of 383 dairy cows from 80 farms were included in this study. All Swiss dairy breeds were represented (Holstein Friesian, Simmental, Red Holstein, Swiss Braunvieh and their crosses), though, primarily Simmental × Red Holstein crosses were present. Data of 163 animals were excluded (total n = 546 cows) because of puerperal endometritis, preceding caesarean section, retained foetal membranes, birth problems and systemic illnesses. Data were collected between May 2001 and April 2002 (12 months).

### Drugs and study design

Cows were distributed to treatment groups A (n = 143), B (n = 127) and C (n = 113).

### Clinical reevaluation

From the 383 cows controlled in the first examination, 61 cows could not be reevaluated. Reasons were insemination in the meantime (9/14/10), selling (1/3/2) or cows were on pasture (8/8/6) allocated in treatment groups A/B/C respectively.

As to side effects reported by owners, there were 2 cows dripping milk for 5 minutes after injection (treatment group A, C) and 1 cow shivering for a couple of minutes in treatment group A.

### Clinical variables

#### Uterine size

Uterine size before drug administration and 14 days later is specified in Table 3.

**Table 3:** Number of cows at first examination 21 to 35 days post partum and 14 days later with uterine size (under 1 hand/pelvic/abdominal-delimitable/abdominal-not delimitable) of treatment groups A, B and C. A is (PGF_2α _+ E2), B is placebo and C is PGF_2α _alone.

**Uterine size**	**First examination**	**Second examination**
**under 1 hand**		
A	58	84
B	47	64
C	49	61
**pelvic**		
A	70	46
B	62	46
C	46	29
**abdominal-delimitable**		
A	11	0
B	13	1
C	9	3
**abdominal-not delimitable**		
A	4	0
B	3	1
C	7	3
		
**Total**	379	336
**Data missing**	4	47

The progress from first to second evaluation was "physiologic" in A = 38%, B = 30% and C = 37% of the cows. There were A = 22%, B = 25% and C = 30% of the cows "healed". The progress was considered "delayed" in A = 40%, B = 45% and C = 33% of the cows. There were no significant differences found among treatment groups, neither all over (p = 0.377) nor divided in "with" or "without corpus luteum".

#### Uterine secretion

Scoring of uterine secretion before drug administration and 14 days later is specified in Table 4.

**Table 4:** Number of cows at first examination 21 to 35 days post partum and 14 days later with uterine secretion (normal/purulent/lochiae) of treatment groups A, B and C. A is (PGF_2α _+ E2), B is placebo and C is PGF_2α _alone.

**Uterine secretion**	**First examination**	**Second examination**
**normal**		
A	132	136
B	108	119
C	87	104
**purulent**		
A	3	3
B	7	1
C	8	3
**lochiae**		
A	8	0
B	11	1
C	16	1
		
**Total**	380	368
**Data missing**	3	15

The progress from first to second evaluation was "physiologic" in A = 91%, B = 88% and C = 76% of the cows. In the group of the healed cows there were A = 7%, B = 10% and C = 20%. "Pathologic" were A = 2%, B = 2% and C = 4% of the cows. There was a significant difference all over (P = 0.019), but only A versus C was significant (P = 0.005). If tests were performed in cows "with" or "without corpus luteum", the result was only significant in "without corpus luteum" (all over P = 0.002; A versus C, P = 0.001).

### Fertility variables (Table 5)

**Table 5:** Number of days from calving to first insemination, number of days open and number of AI until conception for all cows in treatment groups A, B and C.

**Calving to first insemination**	**all cows**	**Group of cows without CL**	**p-value**
A	65 ± 24	62 ± 18	all cows: p = 0.306 without CL: p = 0.034
B	68 ± 23	71 ± 20	
C	64 ± 19	67 ± 16	A-B: p = 0.024* A-C: p = 0.406* B-C: p = 0.953*
**Days open**			
A	95 ± 46	93 ± 44	all cows: p = 0.947 without CL: p = 0.802
B	97 ± 49	98 ± 45	
C	100 ± 53	97 ± 44	
**Number of AI until conception**			
A	1.8 ± 1	1.7 ± 0.9	all cows: p = 0.582 without CL: p = 0.915
B	1.8 ± 1	1.7 ± 1	
C	1.9 ± 1.2	1.7 ± 1	

A is (PGF_2α _+ E2), B is placebo and C is PGF_2α _alone (mean ± SD) for all cows and group of cows without CL. Level of significance is p = 0.05 and with Bonferroni adjustment p < 0.017. * indicates the p-values for pairwise group comparisons of cows without CL.

Interval from calving to first insemination was (mean ± standard deviation) 65 ± 24 in treatment group A, 68 ± 23 in group B and 64 ± 19 in group C. No significant differences were found among treatment groups all over (p = 0.306). In "without corpus luteum", days open were significantly different (p = 0.034) and 10 days shorter for A than for B (p = 0.024).

Days open were (mean ± standard deviation) 95 ± 46 in treatment group A, 97 ± 49 in group B and 100 ± 53 in group C. No significant differences were found among treatment groups, neither all over (p = 0.947) nor divided in "with" or "without corpus luteum".

Number of AI until conception (mean ± standard deviation) was 1.8 ± 1 in treatment groups A and B and 1.9 ± 1.2 in group C. No significant differences were found among treatment groups, neither all over (p = 0.582) nor divided in "with" or "without corpus luteum".

Subsequent treatment of the uterus was performed in 6/3/4 cows in treatment group A, B, C respectively. No significant differences were found among treatment groups, neither all over (p = 0.613) nor divided in "with" or "without corpus luteum".

Subsequent treatment of the ovaries was done in 59/42/40 cows in treatment group A, B, and C, respectively. No significant differences were found among treatment groups, neither all over (p = 0.676) nor divided in "with" or "without corpus luteum".

#### Covariables

Plasma progesterone level on the day of drug administration was (mean ± SD) 3.6 ± 1.3 ng/ml, 3.9 ± 1.8 ng/ml and 3.9 ± 1.6 ng/ml in treatment groups A, B and C "with corpus luteum". Plasma progesterone level on the day of drug administration was (mean ± SD) 0.8 ± 0.4 ng/ml, 0.9 ± 0.4 ng/ml and 0.8 ± 0.3 ng/ml in treatment groups A, B and C without corpus luteum.

## Discussion

We could not corroborate our hypothesis: There was but modest benefit from the use of PGF_2α_, or (PGF_2α _+ E2) on the fertility variables examined. There was a significant difference among the 3 treatment groups in cows without CL, but after Bonferroni adjustment, only a tendency for a shorter interval from calving to first insemination for (PGF_2α _+ E2) as compared to placebo could be demonstrated. No significant effect on the interval from calving to first insemination was found for the cows in the PGF_2α _– group as compared to placebo. This is in contrast to a study by Gay and Upham, where a single injection of PGF_2α _at a median of 25 days post partum reduced median time to first breeding by 4.5 days, but not median time to conception or conception rate in clinically normal cows with a palpable corpus luteum [31]. This fact might be induced by the selection of cows: Gay and Upham only chose cows with a palpable corpus luteum, where PGF_2α _influenced oestrus induction [31]. In our study, the modest benefit of (PGF_2α _+ E2) in cows without a corpus luteum could be explained by a favourable effect on myometrial contractility, which is intensified for (PGF_2α _+ E2) as compared to PGF_2α _alone as shown in experimental diestrus cows [20]. A positive effect of PGF_2α _on cows without corpus luteum as found in our study was already described [32]. Increased uterine contraction and favourable factors involved in uterine defence mechanisms were induced [8,32,33]. In another study, prostaglandin therapy at day 26 and/or 40 independent of cycle state and uterine health provoked a decreased calving to conception interval as compared to saline treated control cows by 19 and 16 days, respectively [34].

Though the cows in our study were judged "healthy" by their owners, there might have been cases of clinical and subclinical endometritis. Rectal palpation and evaluation of vaginal discharge are the basis for diagnosis of subclinical endometritis and possible treatment of most cows in the field [35]. In the cow, a moderate amount of purulent discharge at oestrus or during uterine involution may be part of the self-cleaning process [18]. It is, therefore, difficult to decide whether cows in good general condition but suffering from purulent discharge are healthy (physiologic self-cleaning) or suffering from endometritis. The incidence of post partum uterine infections in clinically healthy cows has been assessed as reducing by self-cure from 92% in the first week to 64% in week 4 and to 25% in week 7 [13]. In a meta-analysis of the prostaglandin effect administered post partum including 4052 cows described in 10 papers, days open were shorter in treated cows as compared to untreated cows (2.6 days) and this difference tended to be greater for cows with an abnormal puerperium (3.3 days) [36].

As the results of our study indicate that the administration of PGF_2α _or (PGF_2α _+ E2) in healthy dairy cows 21 to 35 days post partum had no beneficial effect upon fertility variables, further research will be directed towards the treatment of cows diagnosed with clinical endometritis later than 50 days post partum with PGF_2α_and PGE2 and its effect on reproductive variables.

## Conclusion

The results of this study indicate that the administration of PGF_2α _or a combination of PGF_2α _and PGE2 21 to 35 days post partum had no beneficial effect upon fertility variables measured. This fact is supporting the theory, that blanket treatments in healthy post partum cows should not be performed. A tendency for a shorter interval from calving to first insemination after administration of the combination of PGF_2α _and PGE2, as compared to the placebo group does not justify this therapy. Contrarily, further research should be done in herds with reduced fertility and/or an increased incidence of postpartum vaginal discharge, as the combined prostaglandin treatment may have a positive effect on cycle induction and emptying the uterus of pathological contents.

## Abbreviations

AI = artificial insemination; GnRH = Gonadotropin-releasing hormone; IV = intravenously; PGE2 = prostaglandin E2; PGF_2α _= prostaglandin F_2α _; (PGF_2α _+ E2) = the combination of PGF_2α_and PGE2; CL = corpus luteum

## Competing interests

This study was financially supported by Dr.E. Gräub AG, Berne. Before the beginning of the study, publication of data was bound by contract whatever the results would prove.

## Authors' contributions

GH made substantial contributions to conception and design, acquisition of data, analysis and interpretation of data.

HWB helped with the design and organized financial support of the study.

AS revised the manuscript critically for important intellectual content; and gave final approval of the version to be published.
